# A novel *SPAST* gene splicing variant (c.1617-2A>C) in a heterozygous carrier with hereditary spastic paraplegia

**DOI:** 10.1016/j.ensci.2024.100506

**Published:** 2024-05-29

**Authors:** Elvira Sbragia, Andrea Assini, Silvia Calzavara, Paola Carrera, Claudio Marcello Solaro, Emilio Di Maria

**Affiliations:** a Unit of Neurology, Galliera Hospital, Genoa, Italy; b Department of Neuroscience, Rehabilitation, Ophthalmology, Genetics and Maternal-Child Health, University of Genoa, Genoa, Italy; c Laboratory of Clinical Molecular Genetics, IRCCS Ospedale San Raffaele, Milan, Italy; d Unit of Genomics for Diagnosis of Genetic Diseases, IRCCS Ospedale San Raffaele, Milan, Italy; e Department of Health Sciences, University of Genoa, Genoa, Italy; f University Unit of Medical Genetics, Galliera Hospital, Genoa, Italy

**Keywords:** Hereditary spastic paraplegia, HSP, Spastin, SPG4, *SPAST*, Genotype, VUS, Genetic risk factor, splice variant

## Abstract

Hereditary spastic paraplegia (HSP) is a group of genetically heterogenous neurodegenerative disorders characterized by progressive spasticity and weakness of lower limbs. We report a novel splicing variant (c.1617-2A>C) of the *SPAST* gene in a heterozygous carrier from an Italian family with autosomal dominant HSP. The case study describes a pure form of spastic paraparesis with the cardinal clinical features of SPG4. The novel variant affects a canonical splice site and is likely to disrupt RNA splicing. We conclude that the c.1617-2A>C substitution is a null variant, which could be classified as pathogenic; its penetrance should be further investigated.

## Highlights

• Hereditary spastic paraplegia (HSP) denotes a group of clinically and genetically heterogeneous neurodegenerative disorders; the identification of the underlying pathogenic variant is crucial.
• The case study presents a 59-years-old woman with progressive spasticity and weakness of lower limbs; family history was suggestive for HSP.
• Genetic analysis demonstrated a novel heterozygous variant (c.1617-2A>C) of the *SPAST* gene, which was predicted to disrupt RNA splicing.
• Based on clinical and molecular findings, the c.1617-2A>C substitution could be classified as a pathogenic null variant associated with SPG4.

## 1 Introduction

Hereditary spastic paraparesis (HSP) defines a wide variety of inherited neurological diseases characterized by axonal degeneration of corticospinal tracts and posterior columns [1]. HSP is classified by mode of inheritance (autosomal dominant, autosomal recessive, and X-linked) and whether the primary symptoms occur in isolation or with other neurologic abnormalities. Pure (or “simple”, “uncomplicated”) forms are almost exclusively predominated by slowly progressive spastic paraparesis optionally accompanied by urinary urgency; complicated (or “complex”) forms are associated with several additional symptoms such as intellectual disability or dementia, visual disturbance, seizures, dizziness, dysarthria, dysphagia, ataxia, extrapyramidal signs, muscular atrophy, and peripheral neuropathy [1,2]. Brain magnetic resonance imaging (MRI) might show thin corpus callosum, the so-called “ear-of-the-lynx sign” in some subtypes, enlarged ventricles, bilateral T2-weighted-hypointensity of the globus pallidus and leukoencephalopathy or multifocal areas of white matter T2-weighted-hyperintensities; spinal cord MRI might show global atrophy [3].

HSP represents the second most frequent motor neuron disease with an estimated prevalence of 3–10/100,000 in most populations [2]. To date, over 100 loci/88 genes and 83 different clinical forms were identified in patients with HSP [2]. Pathogenic variants in the *SPAST* (previously known as *SPG4*) gene, encoding the microtubule-severing protein called spastin, cause the most common form of HSP, which is denoted as SPG4. [4].

Based on the autosomal dominant inheritance of most families with HSP, a gain-of-function mechanism has been extensively studied [5]. However, hundreds of null pathogenic variants were found in heterozygous carriers of autosomal dominant HSP, suggesting a loss-of-function (LOF) mechanism. As the spastin protein is a microtubule-severing enzyme, a LOF scenario implies that corticospinal axons degenerate due to inadequate microtubule severing resulting from inactivation of one *SPAST* allele [6].

We describe a patient with a pure autosomal dominant HSP carrying a heterozygous variant (c.1617-2A>C) in the acceptor spice site of intron 14 in the *SPAST* gene. We evaluated the hypothesis that this novel variant is causative of the clinical picture observed in the patient.

## 2 Case report

### 2.1 Family history

**Fig. 1:**
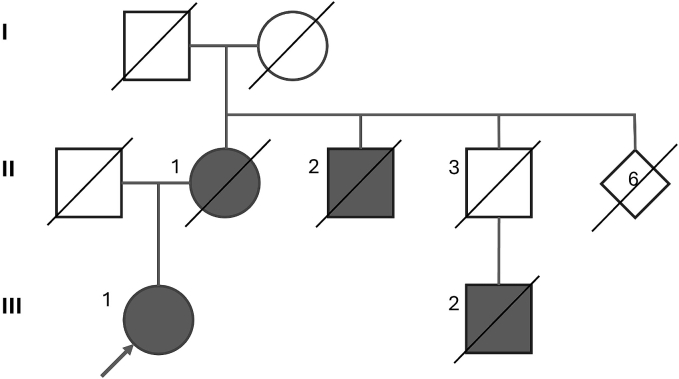
Pedigree of the family. Individuals annotated as affected were clinically diagnosed with spastic paraplegia (II:1, III:1 and III:2), or as suffering from a neurological disorder resembling spastic paraplegia (III:2). The proband of the family (denoted by the arrow) is the heterozygous carrier of the novel c.1617-2A>C variant in the *SPAST* gene described the present report. If not relevant, spouses and gender were omitted to preserve anonymity.

The patient (individual III:1 of the pedigree depicted in Fig. 1) was the only child of non-consanguineous parents, both of Italian ancestry. The mother (II:1, who died at 86 years of age) had been diagnosed as having spastic paraplegia since she was 46 (admission at the Galliera Hospital, Genova, 1969); the diagnosis was further confirmed in a subsequent admission to another hospital in 2001, when the genetic investigations for autosomal dominant spinocerebellar ataxias and prion diseases failed to reveal pathogenic variants. On the maternal side, one uncle (II:2, died at 56) was referred to as walking on his toes since he was young and needing a cane at a later age. Three sibs of the mother (out of 8) were reported as deceased soon after birth. A male cousin (III:2), whose father died due a neoplasm (age at death unknown), was referred to as suffering from an undefined gait disturbance; another female cousin suffers from multiple sclerosis. Both maternal grandparents died after 80 with no apparent movement disorder. Family history did not reveal any other individual with gait disturbances or neurological symptoms. The patient did not have pregnancies.

### 2.2 Clinical features

The patient (female, naturally right-handed) was born at term after uncomplicated pregnancy, labour, and delivery. Physical examination at birth demonstrated head circumference, length, and weight within normal ranges. Developmental milestones were within the normal limits. No delay in her educational history was reported. She currently teaches at a high school. In the past medical history, she reported hypercholesterolemia and glaucoma in both eyes under treatment, bilateral ovariectomy at 56 years of age for ovarian cysts, and operated left nipple melanoma; bone fractures (right arm and left elbow) were due to accidental falls.

At 57 years of age the patient started to complain of subtle left lower limb weakness. Four years later (2019), the symptoms worsened, and the patient underwent specific examinations. Neurological examination, motor evoked potentials (MEP), peripheral sensory and motor electroneurography, electromyography and brain and spine MRI studies did not show abnormalities; one year later MEP showed an increased central conduction time in particular at right side (23.52 milliseconds [msec] at right side and 17.31 msec at left side, normal values [NV] <16.5 msec); distal latencies were normal.

At our first visit (2021), the neurological examination revealed spastic gait, mild bilateral spastic paraparesis, moderate at right lower limb, and diffuse brisk tendon reflexes; no urinary or faecal incontinence was reported; Hoffman sign was negative while Babinski phenomenon resulted positive bilaterally. Brain and spine MRI showed only small non-specific vascular lesions in frontal bilateral white matter; MEP demonstrated severely increased central conduction time at lower limbs, bilaterally and worse at right side (31.7 msec right limb, 24.8 msec left limb, NV <16.5 msec); distal latencies were normal. No signs of cognitive or memory impairment, neither behavioural nor language disturbances were apparent during the clinical sessions. The main characteristics of the case, as compared with a synopsis of the SPG4 cardinal features, are summarised in Table 1.

**Table 1:** Summary of the patient's clinical manifestations, compared to the cardinal features of SPG4 [[1], [2], [3], [4]].

	SPG4 cardinal features	Present case
Inheritance	Autosomal dominant; genetic anticipation, variable clinical manifestation.	Autosomal dominant; possible reduced, age-dependent penetrance.
Clinical presentation	Usually pure slowly progressive spastic paraparesis: lower limb spasticity and weakness, spastic gait, hyperreflexia; variable entity of disability. In some cases, complicated spastic paraparesis with additional symptoms (*e.g.* cognitive impairment, visual disturbance, seizures, ataxia, bulbar symptoms).	Adult-onset pure and isolated paraparesis (worse at right lower limb), spastic gait, hyperreflexia; no cognitive impairment, no additional neurological signs.
Age at onset	Variable, from infancy to >60 yrs.; average 29 ± 17 yrs.	Age at onset 57 yrs.; age at diagnosis 63 yrs.
Neurophysiological alterations	MEP often prolonged, sometimes normal; possible alterations of peripheral nerves	MEP: central motor conduction time (msec) at diagnosis: 31.7 right limb and 24.8 left limb (NV < 16.5); no alterations of peripheral nerves.
MRI alterations	GM and WM lesions; thin corpus callosum; extensive microstructural WM damage; spinal cord atrophy.	Small non-specific vascular lesions in frontal bilateral WM.
Miscellaneous	Comorbidities mostly present in complicated forms as secondary to the associated non-motor symptoms; depression, fatigue, pain, RLS; reduced health quality of life.	Hypercholesterolemia, glaucoma in both eyes, ovarian cysts, left nipple melanoma.
Molecular basis	Missense, nonsense, splice site, insertions, small and large deletions in the *SPAST* gene	Heterozygous c.1617-2A>C splice site variant in the *SPAST* gene

Legend: GM = grey matter, MEP = motor evoked potential, MRI = magnetic resonance imaging, msec = milliseconds, NV = normal values, RLS = restless legs syndrome, WM = white matter, yrs. = years.

### 2.3 Genetic analysis

The patient was enrolled in a structured genetic counselling protocol. After two counselling sessions she provided consent for molecular analysis.

Molecular diagnosis was performed in 2022 by the means of a targeted massive sequencing panel (Illumina NextSeq) including the following genes: *AFG3L2, ALDH18A1, ALS2, AP4B1, AP4E1, AP4M1, AP4S1, AP5Z1, ATL1, ATP13A2, B4GALNT1, BSCL2, C12orf65, CAPN1, CYP2U1, CYP7B1, DDHD1, DDHD2, ERLIN2, FA2H, GBA2, HSPD1, KIF1A, KIF1C, KIF5A, L1CAM, NIPA1, PLP1, PNPLA6, REEP1, REEP2, RTN2, SACS, SPART, SPAST, SPG11, SPG21, SPG7, WASHC5, ZFYVE26*. Relevant variants were confirmed by Sanger sequencing.

The heterozygous NM_014946:c.1617-2A>C variant in the *SPAST* gene was detected and classified as class 4 according to current criteria [7]. No other variant was found. Relatives with definite or possible diagnosis of spastic paraplegia were deceased and could not be investigated.

## 3 Discussion

We reported a patient suffering from lower limb spasticity and weakness, hyperreflexia, impaired corticospinal conduction, with insidious onset and progressive course, and family history consistent with autosomal dominant inheritance, thus resembling most typical features of hereditary spastic paraplegia. The novel c.1617-2A>C variant in the intron 14 of the *SPAST* gene was found in one *SPAST* allele. The comparison of the patient's clinical and electrophysiological features with the cardinal features of SPG4 (Table 1) showed that the patient is affected with a typical “pure” form of spastic paraplegia.

The novel variant was not reported in the gnomAD database of reference populations and should be considered as extremely rare. An adjacent variant, c.1617-3C>T (rs201212542) is reported with frequency < 0.0001 in the European population (source: gnomAD v3.1.2, accessed Sept. 21, 2023).

The c.1617-2A>C substitution affects a canonical splice site (intronic within ±2 of splice site) and is likely to disrupt RNA splicing, thus resulting in an absent or non-functional protein product.

Two single substitutions of the same nucleotide, namely c.1617-2A>T and c.1617-2A>G, were reported as pathogenic in individuals affected with SPG4 [8] (source: NCBI, ClinVar; [VCV000988978.1], https://www.ncbi.nlm.nih.gov/clinvar/variation/VCV000988978.1, [VCV000805512.6], https://www.ncbi.nlm.nih.gov/clinvar/variation/VCV000805512.6, accessed Sept. 21, 2023).

*In silico* algorithms (Splice Site finder – http://www.umd.be/HSF/; Max Ent Scan – http://genes.mit.edu/burgelab/maxent/Xmaxentscan_scoreseq.html; NNSplice - Splice Site Prediction by Neural Network Site – http://www.fruitfly.org/seq_tools/splice.html; Gene Splicer – http://www.cbcb.umd.edu/software/GeneSplicer/gene_spl.shtml) predict that all the 1617-2A>C, A>G and A>T variants may disrupt the acceptor consensus site. Moreover, adjacent single nucleotide substitutions in the same splice site (c.1617-1G>A and c.1617-1G>T) were *in silico* predicted as pathogenic [9]. Though no functional evidence is available, this suggests that the acceptor splice site in intron 14 is crucial for normal RNA splicing, and that abnormal splicing in this region may lead to HSP phenotypes. Thus, the c.1617-2A>C substitution may be considered as a null variant.

Loss-of-function (LOF) is a known mechanism of SPG4: to date, the list of *SPAST* variants reports 43% truncating variants; among these variants, 14% are reported in splice junctions and are not clustered in particular regions of the gene (source: LOVD, https://databases.lovd.nl/shared/genes/SPAST, accessed May 3, 2024). Thus, in comparison with the *SPAST* splice site pathogenic variants previously reported, c. 1617-2A>C does not appear to be located in a hot spot [10].

Several observations supported the hypothesis that the c.1617-2A>C substitution is associated with SPG4. Conversely, other clues are lacking – most important, the variant segregation within the family could not be investigated, as our patient is the only affected member of the family who underwent full clinical and genetic examination; similarly with other late-onset neurodegenerative disorders, investigations in previous generations were not applicable. However, assuming the c.1617-2A>C variant as the disease-causing, the family history reported herein suggests that reduced, age-dependant penetrance should be considered. Previous studies showed an age-dependent penetrance <1, with lower estimates in females [11].

In summary, we described a patient with pure isolated spastic paraplegia consistent with the SPG4 form, which was inherited as an autosomal dominant trait; the genetic analysis revealed the novel c.1617-2A>C variant in the *SPAST* gene, which affects a canonical splice site and is likely to disrupt RNA splicing. We would conclude that the *SPAST* variant is responsible for the clinical picture observed in the present case and could be classified as a novel pathogenic variant associated to SPG4. Further observations are needed to ascertain its biological role.

## Patient's consent

This case study was conducted retrospectively from data obtained for clinical purposes. The patient explicitly agreed to publish the present report. All relevant data were treated according to current rules and recommended best practice.

## Availability of data and materials

Data sharing not applicable to this article as no datasets were generated.

## Funding statement

This research did not receive any specific grant from funding agencies in the public, commercial, or not-for-profit sectors.

## Authorship

All authors materially participated in the research and article preparation. All authors have approved the final manuscript.

## CRediT authorship contribution statement

**Elvira Sbragia:** Writing – original draft, Investigation, Conceptualization. **Andrea Assini:** Writing – review & editing, Conceptualization. **Silvia Calzavara:** Investigation. **Paola Carrera:** Writing – original draft. **Claudio Marcello Solaro:** Writing – review & editing. **Emilio Di Maria:** Writing – review & editing, Writing – original draft, Conceptualization.

## Declaration of competing interest

None to declare.
